# Inhaled Corticosteroids Selectively Alter the Microbiome and Host Transcriptome in the Small Airways of Patients with Chronic Obstructive Pulmonary Disease

**DOI:** 10.3390/biomedicines10051110

**Published:** 2022-05-11

**Authors:** William Yip, Xuan Li, Graeme J. Koelwyn, Stephen Milne, Fernando Sergio Leitao Filho, Chen Xi Yang, Ana I. Hernández Cordero, Julia Yang, Cheng Wei Tony Yang, Tawimas Shaipanich, Stephan F. van Eeden, Janice M. Leung, Stephen Lam, Kelly M. McNagny, Don D. Sin

**Affiliations:** 1 Centre for Heart Lung Innovation, The University of British Columbia, St. Paul’s Hospital, Vancouver, BC V6Z 1Y6, Canada; willyip@brc.ubc.ca (W.Y.); annie.li@hli.ubc.ca (X.L.); graeme.koelwyn@hli.ubc.ca (G.J.K.); stephen.milne@hli.ubc.ca (S.M.); fernando.studart@hli.ubc.ca (F.S.L.F.); yolanda.yang@hli.ubc.ca (C.X.Y.); ana.hernandez@hli.ubc.ca (A.I.H.C.); julia.yang@hli.ubc.ca (J.Y.); tony.yang@hli.ubc.ca (C.W.T.Y.); tshaipanich@providencehealth.bc.ca (T.S.); stephan.vaneeden@hli.ubc.ca (S.F.v.E.); janice.leung@hli.ubc.ca (J.M.L.); sclam@mail.ubc.ca (S.L.); kelly@brc.ubc.ca (K.M.M.); 2 School of Biomedical Engineering, Faculties of Medicine and Applied Sciences, The University of British Columbia, St. Paul’s Hospital, Vancouver, BC V6T 1Z3, Canada; 3 Division of Respiratory Medicine, Department of Medicine, The University of British Columbia, Vancouver, BC V5Z 1M9, Canada; 4 Faculty of Health Sciences, Simon Fraser University, Burnaby, BC V5A 1S6, Canada; 5 British Columbia Cancer Agency, The University of British Columbia, Vancouver, BC V5Z 4E6, Canada

**Keywords:** COPD, inhaled corticosteroids, 16S rRNA gene sequencing, mRNA-sequencing, transcriptomics, bronchoscopy, fluticasone, budesonide, microbiome, inflammation

## Abstract

Background: Patients with chronic obstructive pulmonary disease (COPD) are commonly treated with inhaled corticosteroid/long-acting ß2-agonist combination therapy. While previous studies have investigated the host–microbiome interactions in COPD, the effects of specific steroid formulations on this complex cross-talk remain obscure. Methods: We collected and evaluated data from the Study to Investigate the Differential Effects of Inhaled Symbicort and Advair on Lung Microbiota (DISARM), a randomized controlled trial. Bronchoscopy was performed on COPD patients before and after treatment with salmeterol/fluticasone, formoterol/budesonide or formoterol-only. Bronchial brush samples were processed for microbial 16S rRNA gene sequencing and host mRNA sequencing. Longitudinal changes in the microbiome at a community, phylum and genus level were correlated with changes in host gene expression using a Spearman’s rank correlation test. Findings: In COPD patients treated with salmeterol/fluticasone, the expression levels of 676 host genes were significantly correlated to changes in the alpha diversity of the small airways. At a genus level, the expression levels of 122 host genes were significantly related to changes in the relative abundance of *Haemophilus*. Gene enrichment analyses revealed the enrichment of pathways and biological processes related to innate and adaptive immunity and inflammation. None of these changes were evident in patients treated with formoterol/budesonide or formoterol alone. Interpretation: Changes in the microbiome following salmeterol/fluticasone treatment are related to alterations in the host transcriptome in the small airways of patients with COPD. These data may provide insights into why some COPD patients treated with inhaled corticosteroids may be at an increased risk for airway infection, including pneumonia. Funding: The Canadian Institute of Health Research, the British Columbia Lung Association, and an investigator-initiated grant from AstraZeneca.

## 1. Introduction

Inhaled corticosteroids (ICS) and long-acting ß2-agonists (LABA) are commonly prescribed medications for patients with moderate-to-severe chronic obstructive pulmonary disease (COPD) [1]. Compared with LABA monotherapy, combination therapy with ICS/LABA has been shown to improve health status and lung function and reduce the risk of acute exacerbations [1]. However, several studies have shown that COPD patients treated with ICS and, in particular, inhaled fluticasone are at an increased risk of pneumonia [2,3]. While not fully settled, the risk of pneumonia in this setting may in part relate to the steroid formulation and potency, with lipophilic and potent steroids enhancing the risk [2,4,5,6].

The risk of pneumonia may also relate to the airway microbiome, which is perturbed in COPD. We have recently shown that ICS/LABA use is associated with a relative reduction in alpha diversity of the small airway microbiome in COPD [7]. Here, we hypothesized that ICS therapy is associated with changes in the airway microbiome that are related to host transcriptomic changes in the small airway microenvironment. We also hypothesized that these changes are steroid-specific based on potency and lipophilicity. To investigate these hypotheses, we applied bacterial 16S rRNA gene sequencing and host mRNA-sequencing to bronchial brushes to obtain profiles of the small airway microbiome and host transcriptome from COPD patients treated with different ICS/LABA combinations. 

## 2. Materials and Methods

### 2.1. Study Cohort and Design

**Figure 1:**
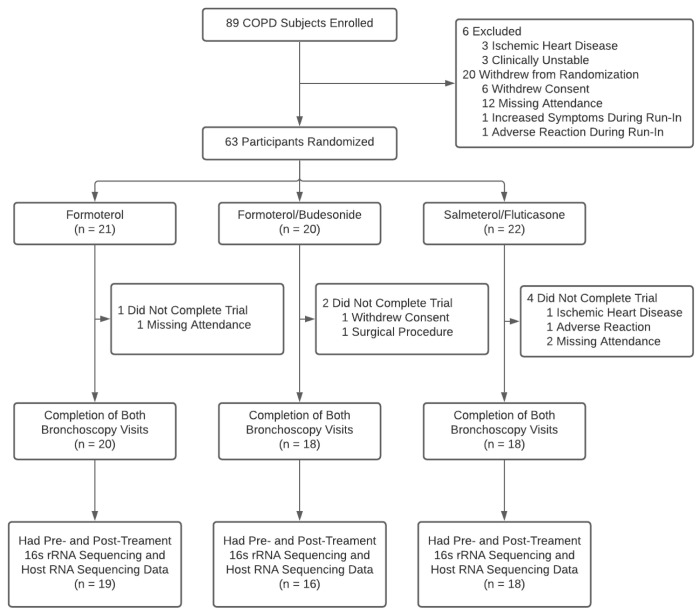
Participant flow diagram. Abbreviations: COPD, chronic obstructive pulmonary disease.

We used data from the Study to Investigate the Differential Effects of Inhaled Symbicort and Advair on Lung Microbiota (DISARM), which was a randomized controlled trial aimed at evaluating the effects of inhaled fluticasone and budesonide on the airway microbiome of patients with COPD. The DISARM trial is registered at ClinicalTrials.gov under the identifier NCT02833480 and has approval from the University of British Columbia/Providence Health Care Research Ethics Committee (H14-02277). The patient cohort and overall study design of the DISARM trial have been previously described in detail and are also shown in Figure 1 [7].

Briefly, a total of 89 COPD patients who were clinically stable for at least 8 weeks and did not receive prior treatment with systemic corticosteroids and/or antibiotics were enrolled in the study. After providing informed consent, all participants underwent a 4-week run-in period during which they discontinued ICS (if they were on them) and received only formoterol (Oxeze Turbuhaler^®^ 12 ug twice daily) as a maintenance therapy to wash out any residual effects of ICS in the lower airways. At the end of the 4-week run-in period, participants underwent a baseline bronchoscopy where bronchial brushes were obtained from the 6th to 8th generation airways of the right upper lobe (or left upper lobe if the right upper lobe was not accessible) using a steel-tipped cytology brush. Following the baseline bronchoscopy, 63 participants were randomly assigned to three treatment groups for 12 weeks: salmeterol/fluticasone (SAL/FLU; Advair Diskus^®^ 250 ug twice daily; *n* = 22), formoterol/budesonide (FOR/BUD; Symbicort Turbuhaler^®^ 400 ug twice daily; *n* = 20) and formoterol-only (FOR; Oxeze Turbuhaler^®^ 12 ug twice daily; *n* = 21). The FOR group was considered the control group. At the end of the 12-week treatment period, participants underwent a follow-up bronchoscopy where bronchial brushes were obtained from the same airway as where the first bronchoscopic sampling took place. 

### 2.2. Microbial DNA Extraction and PCR Amplification

Bronchial brushings for microbiome profiling were collected and stored in Cytolyt (Hologic, Marlborough, MA, USA) for DNA preservation as previously described [7]. Methods for microbial DNA extraction and PCR amplification have been described in detail previously [7]. DNA was extracted from cytological brush specimens using the DNeasy Blood and Tissue Kit (QIAGEN, Toronto, ON, Canada) according to the manufacturer’s instructions. DNA extraction was followed by PCR amplification using primers targeting the V4 region of the 16S rRNA gene. Purified PCR products were sequenced using the Illumina MiSeq platform (Illumina, San Diego, CA, USA). 

### 2.3. Microbiome Profiling

Methods for profiling the microbiome have been described in detail previously [7]. In brief, raw sequencing reads (fastq files) were processed using the Quantitative Insights into Microbial Ecology (QIIME2, version 2020.2) [8]. The divisive amplicon denoising algorithm (DADA2) was used to denoise and cluster the sequencing reads into amplicon sequence variants (ASVs) and merge the paired-end reads [9]. A Naïve Bayes taxonomic classifier was trained on the SILVA rRNA database (v132, Ref NR 99) and used for taxonomic assignment [10,11]. Low abundant taxa and ASVs with no taxa annotation at a phylum level were omitted from the downstream analysis.

### 2.4. Host RNA Extraction and RNA Sequencing 

Bronchial brushings for host transcriptomic profiling were collected and stored in QIAzol RNA lysis buffer (QIAGEN, Stockach, Germany) to preserve RNA prior to extraction. RNA was extracted from cytological brush specimens using the RNeasy Plus kit (QIAGEN, Stockach, Germany) according to the manufacturer’s protocol and sequenced at the Biomedical Research Centre Sequencing Core at the University of British Columbia. Sample quality control was performed using the Agilent 2100 Bioanalyzer (Agilent, Santa Clara, CA, USA). Qualifying samples were then prepared following the standard protocol for the NEBnext Ultra ii Stranded mRNA (New England Biolabs, Ipswich, MA, USA). mRNA Sequencing was subsequently performed on the Illumina NextSeq500 (Illumina, San Diego, CA, USA) platform with paired end 42 bp × 42 bp reads, as previously described [12].

### 2.5. Host Transcriptomic Profiling

Raw sequencing reads were analyzed for quality control using FastQC [13]. Salmon was used for quantification and quasi-alignment of the sequencing reads to the GENCODE genome reference (assembly GRCh37, release 31) [14,15]. Transcript level counts and transcripts per million (TPM) were summarized to gene level using the “tximport” R package, and gene level counts were filtered using the “filterByExpr” function in the “edgeR” package [16,17]. The R package “limma” was used to normalize the count data to log_2_ counts per million (CPM) [18,19]. After genes with low abundance (log_2_CPM < 1 or TPM < 2 in more than 80% of the samples) were filtered out, a total of 15,667 genes remained. 

### 2.6. Statistical Analysis

Longitudinal changes in the microbiome as well as the expression levels of individual genes were quantified as post- minus pre-treatment. Only participants (*n* = 53) with both pre- and post-treatment gene expression data as well as microbiome data were included in the current study. From these participants, we independently correlated changes in gene expression (∆log_2_CPM) in each treatment group with changes in alpha diversity (Shannon diversity index) [20], beta diversity (Unweighted UniFrac Distance Matrix) [21] and relative abundances of the main taxa (at the phylum and genus levels) using Spearman’s rank correlations. Statistical significance was set at a Benjamini–Hochberg false discovery rate (FDR) of less than 0.1. From the significantly correlated genes, the ingenuity pathway analysis (IPA) (QIAGEN, Redwood City, CA, USA) was used to identify top canonical pathways, enriched biological pathways and diseases, and upstream transcription, cytokine and transmembrane receptor regulators. To further validate the enriched pathways identified by IPA, we performed pathway enrichment analyses using the package WebGestaltR [22] in R (version 4.0.5) [23] on the same set of genes using gene ontology (GO) terms and the Kyoto Encyclopedia of Genes and Genomes (KEGG) pathways [24,25]. 

## 3. Results

### 3.1. Study Cohort

Fifty-three participants had both pre-and post-treatment gene expression data as well as microbiome data (Figure 1). Baseline characteristics are shown in Table 1. No significant differences according to demographics, lung function and clinical data were detected across the three treatment groups (*p* > 0.05).

### 3.2. Changes in Alpha Diversity of the Airway Microbiome Are Related to Changes in Host Gene Expression in the Small Airways

To investigate the host–microbiome relationship in the small airways of COPD patients treated with FOR/BUD or SAL/FLU, we compared the change (post- minus pre- treatment) in the microbiome at a community, phylum, and genus level with changes in host gene expression. 

**Figure 2:**
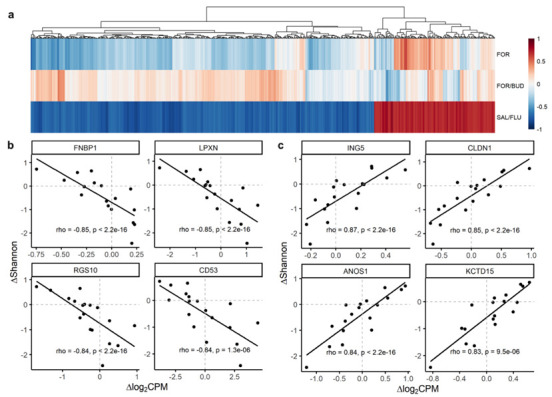
Changes in gene expression in the small airways are related to changes in the microbiome at a community level. (**a**) Heat map depicting significant (FDR < 0.1) correlations between changes (post- minus pre-treatment) in host cell gene expression (delta log_2_ counts per million (∆log_2_CPM)) and changes in the Shannon diversity index (∆Shannon) in at least one of the three treatment groups: formoterol-only (FOR), formoterol/budesonide (FOR/BUD) or salmeterol/fluticasone (SAL/FLU). Colour represents Spearman’s correlation coefficient. Columns represent individual genes and are arranged using hierarchical clustering. (**b**) Scatterplots of select genes that are negatively correlated. (**c**) Scatterplots of select genes that are positively correlated. Abbreviations: FDR, false discovery rate.

Using a Spearman’s rank correlation, we related changes in alpha diversity using the Shannon index as a summary indicator with changes in host gene expression in each of the three treatment groups. Of the 15,667 genes analyzed, we noted changes in the expression levels of 676 genes (500 negative and 176 positive) in COPD patients treated with SAL/FLU and these correlated significantly (FDR < 0.1) with changes in the Shannon index (Figure 2). At a more stringent threshold of FDR < 0.05, 180 genes remained significantly correlated with changes in the Shannon index in the SAL/FLU group. Of the 676 genes significantly correlated with changes in the Shannon Index, the majority were hematopoietic and inflammatory cell-linked, followed by mesenchymal/stromal- and epithelial-linked genes. Interestingly, out of the 15,667 genes analyzed, no genes were related to changes in the Shannon index in the FOR/BUD or FOR treatment group. In all three treatment groups, changes in beta diversity did not correlate significantly with changes in host gene expression (data not shown).

### 3.3. Changes in Host Gene Expression Are Related to Phylum Level Changes in the Small Airway Microbiome

Next, we investigated the relationship between changes in the relative abundance of airway microbes at a phylum level and changes in genes expressed by cells in the small airway microenvironment. We independently correlated the changes in the relative abundance of phylum groups with the changes in host gene expression in each of the treatment groups. Of the 18 phyla detected in the microbiome samples, the most abundant were Firmicutes (mean relative abundance 39.8%) followed by Bacteroidetes (24.3%), Proteobacteria (20.7%), Actinobacteria (10.3%), and Fusobacteria (2.45%), comprising approximately 98% of the sequencing reads. The changes in the relative abundance of these top five most abundant phyla were correlated with changes in host gene expression. Statistically significant (FDR < 0.1) genes are shown in Figure S1. The SAL/FLU treatment group had the highest number of genes showing a correlation with relative abundance data at the phylum level: Firmicutes (*IGFBP3*), Bacteroidetes (*LPAR6*), Actinobacteria (*KLRC1*), and Fusobacteria (14 genes). In the FOR/BUD treatment group, only *DONSON* was significantly correlated with the change in the relative abundance of Fusobacteria. Lastly, in the FOR treatment group, *GGA3*, *WDR92*, *EIF3J-DT*, *ADAM28* and *SPATA5L1* were correlated with the change in the relative abundance of Bacteroidetes. 

### 3.4. Changes in Host Gene Expression Are Related to Genus Level Changes in the Small Airway Microbiome

**Figure 3:**
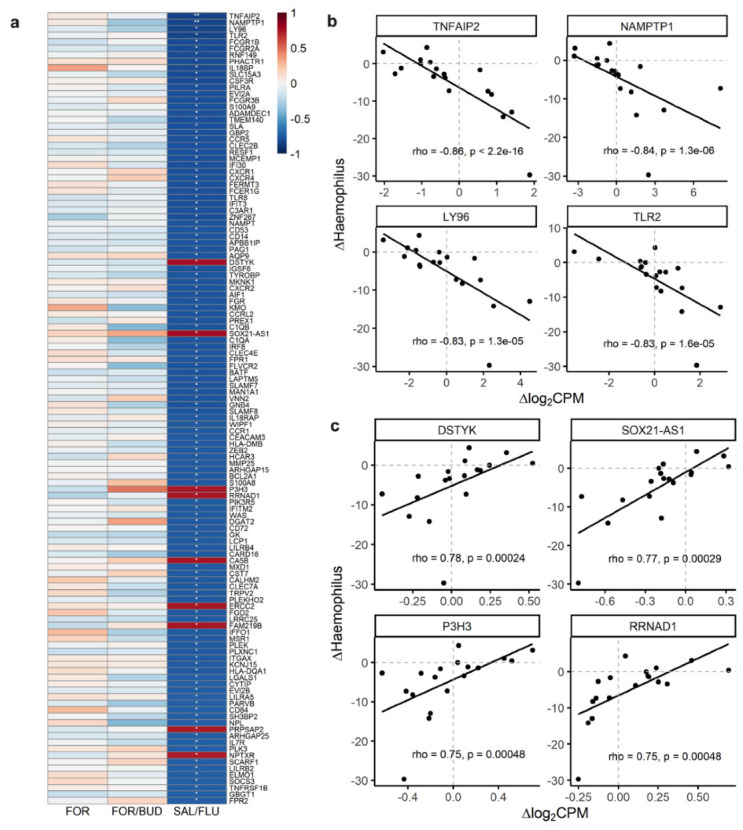
Changes in gene expression in the small airways are related to changes in the relative abundance of Haemophilus. (**a**) Heat map depicting significant correlations between changes (post-minus pre-treatment) in host cell gene expression (delta log_2_ counts per million (∆log_2_CPM)) and changes in the relative abundance of Haemophilus in at least one of the three treatment groups: formoterol-only (FOR), formoterol/budesonide (FOR/BUD) or salmeterol/fluticasone (SAL/FLU). Colour represents Spearman’s correlation coefficient. Rows represent individual genes. ** represents an FDR < 0.05. * represents an FDR < 0.1. (**b**) Scatterplots of select genes that are negatively correlated. (**c**) Scatterplots of select genes that are positively correlated. Abbreviations: FDR, false discovery rate.

We investigated the relationship between microbes at a genus level and host cells in the small airway following ICS/LABA treatment. We independently related changes in the relative abundance of airway microbes at a genus level with changes in host gene expression in each of the three treatment groups. Of the detected genera, the five most abundant were *Streptococcus* (mean relative abundance 19.8%) followed by *Prevotella* 7 (13.3%), *Veillonella* (12.6%), *Haemophilus* (7.7%), and *Rothia* (3.8%), comprising approximately 59% of the sequencing reads. The changes in the relative abundance of these five genera were related to changes in the host gene expression. Statistically significant (FDR < 0.1) genes are shown in Figure S2. We identified 122 genes, primarily hematopoietic and inflammatory genes, that were significantly correlated with changes in the relative abundance of *Haemophilus* in the SAL/FLU treatment group (Figure 3). Of the 122 genes identified, 113 genes were negatively correlated, and nine genes were positively correlated. In addition, the change in the relative abundance of Streptococcus was correlated with the change in the expression of *FBXL6*, *FHAD1*, *SLC25A21-AS1*, *AC093495.1* and *ZNF704* in the SAL/FLU treatment group. Conversely, only *ZNF236-DT* from the FOR treatment group was correlated with the change in the relative abundance of *Rothia,* and no genes showed any significant correlation in the FOR/BUD treatment group. 

### 3.5. Salmeterol/Fluticasone Treatment Is Associated with Enriched Biological Pathways in the Small Airways 

**Figure 4:**
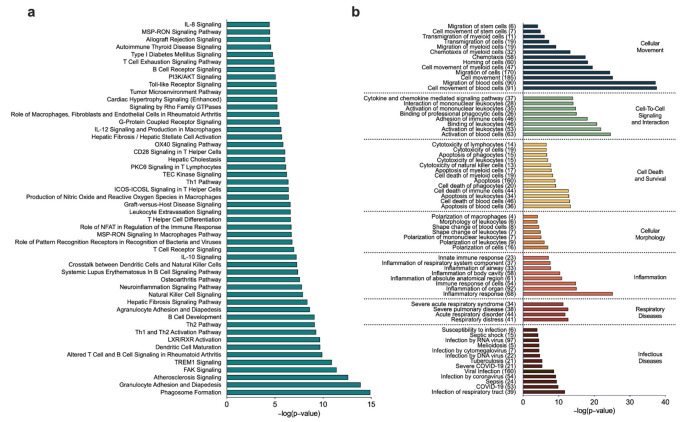
Ingenuity pathway analysis of the 676 host cell genes significantly correlated (FDR < 0.1) with changes in the Shannon diversity index in patients with chronic obstructive pulmonary disease treated with salmeterol/fluticasone. (**a**) Top 50 canonical pathways of genes significantly correlated to changes in the Shannon diversity index. (**b**) Enriched biological functions, cellular processes and diseases. The number in the parentheses represents genes from our 676 gene dataset that overlap with genes that appear in the enriched biological function, cellular process or disease.

Next, we explored the functional role of the significantly correlated genes to gain insights into how they may be involved in the complex interactions between the small airway microbiome and host cells in the small airway microenvironment. We first performed enrichment analysis using IPA followed by validation with GO terms and KEGG pathways. Of the previous analyses that generated gene lists, only resulting gene lists that were larger than 20 significant genes were evaluated for pathway enrichment. From the 676 genes, which were significantly related to changes in the Shannon Index in the SAL/FLU treatment group, we identified 176 canonical pathways that were significantly (*p* < 0.05) enriched. The top 50 canonical pathways are shown in Figure 4a. Interestingly, these included pathways involving innate and adaptive immunity, extracellular and intracellular signaling, cell adhesion, chemotaxis and migration of immune cells, cellular cytoskeletal reorganization, and metabolic and biochemical processes. In addition, we identified significantly (*p* < 0.05) enriched biological functions and cellular processes related to cellular movement, cell-to-cell signaling and interaction, cell death and survival, cellular morphology, and inflammation (Figure 4b). To further identify factors potentially involved in regulating the 676 significantly correlated genes, we performed upstream regulator analysis using IPA, which predicts potential upstream regulators, including transcription factors, cytokines, and transmembrane receptors demonstrated experimentally to alter the affected gene pathways (Figure S3a). The top five upstream cytokine regulators included *IFNG*, *CSF2*, *IL13*, *IL17A*, and *IL10*. Top five upstream transcription and transmembrane receptor regulators included *CEBPA*, *SMARCA4*, *RELA*, *ECSIT*, *STAT3* and *TREM1*, *TLR2*, *CD40*, *TLR7*, *TNFRSF18,* respectively. Subsequent additional pathway enrichment analyses using GO revealed 141 biological processes that were significantly (FDR < 0.05) enriched. The top 50 GO pathways with the greatest percentage of overlapping genes, which, similar to IPA, showed primarily immunological processes, are shown in Figure S4a. From the KEGG enrichment analysis, we identified 44 pathways that were significantly enriched (Figure S4b). Similar to the GO pathways identified, immune pathways and biological processes related to the activation of the innate and adaptive immune response were primarily enriched followed by pathways related to infectious diseases (e.g., *Staphylococcus aureus* infection). 

**Figure 5:**
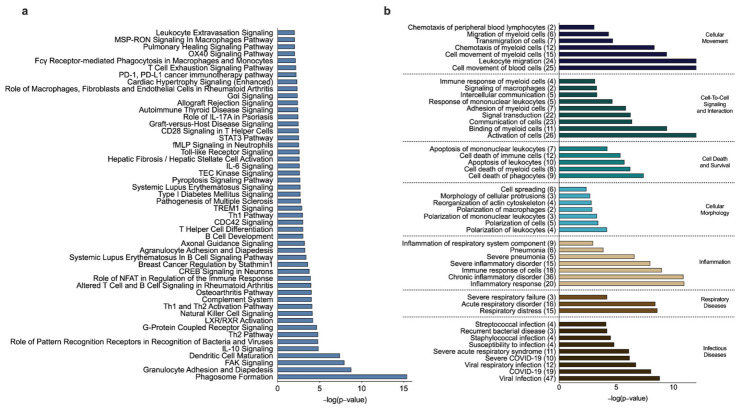
Ingenuity pathway analysis of the 122 host cell genes significantly correlated (FDR < 0.1) with changes in the relative abundance of Haemophilus in patients with chronic obstructive pulmonary disease treated with salmeterol/fluticasone. (**a**) Top 50 canonical pathways of genes significantly correlated to changes in the relative abundance of Haemophilus. (**b**) Enriched biological functions, cellular processes and diseases. The number in the parentheses represents genes from our 122 gene dataset that overlap with genes that appear in the enriched biological function, cellular process or disease.

From the 122 genes that were related to the changes in the relative abundance of Haemophilus in the SAL/FLU treatment group, we identified 94 canonical pathways that were significantly (*p* < 0.05) enriched, the majority of which were pathways related to innate and adaptive immunity. The top 50 canonical pathways are shown in Figure 5a. Biological functions and cellular processes enriched from the 122 genes are shown in Figure 5b. Upstream cytokine, transcription and transmembrane receptor regulators are shown in Figure S3b. The top five upstream cytokine regulators included *IFNG*, *IL13*, *IL10*, *CSF2* and *IL17A*. Top five upstream transcription and transmembrane receptor regulators included *STAT1*, *CEBPA*, *STAB1*, *STAT3*, *CEBPB* and *TLR2*, *CD69*, *CD14*, *TNFRSF10A*, *CD40,* respectively. Pathway enrichment analyses using GO revealed 45 biological processes that were significantly (FDR < 0.05) enriched, the majority of which were pathways related to innate and adaptive immunity. These included macrophage activation, T cell activation, cell chemotaxis, interleukin-12 production, granulocyte activation, neutrophil-mediated immunity, and antigen processing and presentation (Figure S5a). From the KEGG enrichment analysis, we identified eight pathways at FDR < 0.05, and the top 10 pathways are shown in Figure S5b. 

## 4. Discussion

To our knowledge, this study is the first to explore the complex relationship between the airway microbiome and host transcriptome of small airways in COPD patients treated with ICS using data from a randomized controlled trial (RCT). Previous large RCTs have shown that inhaled fluticasone-based therapy increases the risk of pneumonia by ~75% (compared with LABA alone or placebo) in patients with COPD [26]. On the other hand, the use of budesonide, especially in low doses, has not been associated with pneumonia in COPD [27]. Moreover, there is growing evidence that dysbiosis (and, in particular, a reduction in microbial diversity) is a significant contributor to the occurrence of severe pneumonia [28]. While previous studies have investigated the relationship between the lung microbiome and host transcriptome in COPD [29,30,31], the effects of specific steroid formulations on this complex cross-talk have not been adequately explored. To address this knowledge gap, our study compared the effects of SAL/FLU and FOR/BUD on the changes in the microbiome and host transcriptome in the small airways of patients with COPD. Here, we show that in COPD patients treated with SAL/FLU, the host gene expression levels in the small airways are significantly related to longitudinal changes in their microbiome at a community, phylum and genus level. Specifically, longitudinal changes in the alpha diversity and the relative abundance of *Haemophilus* were primarily associated with changes in the expression levels of genes commonly found in hematopoietic and inflammatory immune cells among patients treated with SAL/FLU. Furthermore, changes in alpha diversity were also associated with expression level changes in certain epithelial and mesenchymal/stromal cell genes. Similar trends were not observed in the FOR/BUD or FOR treatment group, suggesting that these observations are steroid-specific and may be, in part, related to the more potent and lipophilic nature of fluticasone compared to budesonide [6].

There is a growing body of evidence that suggests ICS can alter the airway microbiome in COPD patients, but cellular responses in the host that are related to airway dysbiosis are poorly understood. Our findings support the notion that ICS therapy is tightly linked to alterations in gene expression and microbiome changes in the small airway microenvironment. Consistent with our observations, several studies have demonstrated an association between the lung microbiome and host gene transcriptome in COPD lung tissue [29], sputum [30] and bronchial brushings [31]. In lung tissue, we have previously demonstrated an association between the Shannon Index and host gene expression, which is consistent with our current findings [29]. Among the major genera analyzed in the present study, *Haemophilus* was the most strongly associated with changes in genes related to the innate and adaptive immune response in COPD patients, and this finding was identified exclusively in the SAL/FLU group. This is consistent with the findings by Wang and colleagues, who demonstrated in sputum samples an association between *Haemophilus* and the host immune transcriptome during both clinical stability as well as during COPD exacerbations [30]. In contrast, our findings are slightly different from those reported by Ramsheh and colleagues [31]. These authors found that Prevotella and Moraxella were associated with expression level changes in genes involved in immunity and inflammation in COPD patients treated with ICS. However, this study was not an RCT, and there was no run-in period wherein ICS was discontinued before obtaining bronchial brush samples. In addition, they did not analyze the effects of specific steroid formulations, which could influence the microbial composition and/or transcriptomic patterns. Nonetheless, our study extends their findings by exploring the effects of different ICS formulations and using RCT methodology.

Our findings support the view that ICS use, particularly fluticasone, is associated with changes in both the microbiome as well as the transcriptome of hematopoietic and immune cells in the small airways. However, whether changes in the airway microbiome precede host gene expression changes or are a consequence of altered gene expression related to ICS use cannot be concluded from the findings of this study. Our findings likely represent one of three possible scenarios. With regards to the first scenario, ICS may directly alter the airway microbiome, which then may have indirect effects on gene expression changes in cells in the local airway microenvironment. Whether ICS can directly interact with bacteria via ligand–receptor interactions in the airway microbiome has not yet been explored; however, it has been reported that bacteria possess receptors for, and respond to, several mammalian hormones [32]. In addition, androgens and glucocorticoids have been shown to impact microbial growth by affecting the rate of doubling of both Gram-positive and Gram-negative bacteria [32]. Although the supporting evidence is not conclusive, it is reasonable to hypothesize that ICS directly alters the airway microbiome, leading to dysbiosis. Indirect effects on host hematopoietic immune cells may then be facilitated by signals provided by the dysbiotic microbiome in the form of bacterial metabolites. More recently, it has been shown that metabolically active microbes within the small airway microbiome are capable of producing immunomodulatory metabolites such as short-chain fatty acids (SCFAs) [33]. The role of SCFAs in modulating the systemic immune response in various chronic inflammatory lung diseases has been studied extensively [34,35,36,37,38,39,40]. SCFAs produced by the small airway microbiome may modulate the local immune response by similar mechanisms as gut-derived SCFAs. As receptors for SCFAs are abundantly expressed on both immune cells [40,41] and epithelial cells [42], gene expression change as a consequence of ICS-driven microbial dysbiosis in COPD is certainly a possibility. Second, contrary to the previous scenario, ICS may directly interact with steroid receptors expressed in cells in the local surrounding airway, particularly immune cells, which may have downstream transcriptomic effects on inflammatory genes that lead to indirect changes to the resident microbiota. Consistent with this notion, a previous study has shown that ICS treatment induces microbial dysbiosis in the airways by suppressing cathelicidin produced by the airway epithelium [43]; however, our data did not demonstrate an association between changes in the microbiome and changes in the genes associated with cathelicidin. Nonetheless, the broad suppression of the immune response by corticosteroids may allow for certain pathogenic strains of bacteria to colonize the airway resulting in microbial dysbiosis [44]. Finally, while our study suggests that changes in the microbiome are related to changes in the hematopoietic compartment of the small airways or vice versa, these two variables may be affected by ICS independently and simultaneously. The effects of ICS on the host–microbiome relationship are complex and will require future studies to clarify the directionality of this cross-talk.

There were several important limitations to the current study. First, the sample size was relatively small. This was in part due to challenges in (1) performing two bronchoscopy procedures per subject and (2) discontinuing ICS during the run-in phase for some patients. Second, we recruited clinically stable patients with moderate-to-severe COPD. As such, the findings from this study cannot be extended to patients with milder diseases and may not be applicable to those who experience acute exacerbations. Third, the findings reported in this study are relational and do not demonstrate causality or directionality. Lastly, our study did not investigate the proteome, metabolome or epigenome of the small airway because it was beyond the purview of the original RCT. Applying a multi-omics approach would allow for a deeper comprehensive analysis of the small airway environment, which may provide clarity on the mechanistic effects of ICS on the host–microbiome interaction in the context of ICS-driven pneumonia in COPD. 

In summary, our data indicate that transcriptomic changes in (chiefly immune) cells in the small airway microenvironment are related to changes in the airway microbiome, specifically the alpha diversity and relative abundance of the *Haemophilus* genus, in COPD patients treated with salmeterol/fluticasone. Our findings provide insights into the dynamic host–microbiome relationship that may be important in identifying COPD patients who may be at increased risk of pneumonia. 

## Figures and Tables

**Table 1:** Baseline characteristics of participants included in the study (*n* = 53). Table shows the median and interquartile range for quantitative variables. Abbreviations: BMI, body mass index; Post-BD FEV_1_, post-bronchodilator forced expiratory volume in one second; FVC, forced vital capacity; ICS, inhaled corticosteroids.

Variables	FOR *n* = 19	FOR/BUD *n* = 16	SAL/FLU *n* = 18
Age, years	63.0 (56.0–67.0)	66.0 (62.5–70.3)	66.0 (60.3–72.8)
Male, *n* (%)	18 (94.7%)	14 (87.5%)	14 (77.8%)
BMI, kg/m^2^	24.4 (22.0–31.3)	23.9 (19.4–28.5)	28.0 (23.2–32.6)
Current smokers, *n* (%)	10 (52.6%)	6 (37.5%)	10 (55.6%)
Smoking exposure, pack years	45.0 (22.0–60.0)	51.5 (43.3–58.5)	48.0 (34.5–59.0)
Post-BD FEV_1_, % of predicted	66.3 (50.0–73.5)	58.0 (48.1–70.5)	61.5 (47.9–86.5)
Post-BD FEV_1_/FVC, %	56.1 (49.6–62.7)	49.0 (44.6–59.2)	63.1 (49.9–66.7)
Hypertension, *n* (%)	4 (21.1%)	5 (31.3%)	3 (18.8%)
ICS at enrollment, *n* (%)	12 (63.2%)	7 (43.8%)	10 (62.5%)

## Data Availability

Microbiome sequencing data used in this study can be found publicly on the National Center for Biotechnology Information’s Sequence Read Archive (SRA) under the BioProject number PRJNA685554. Gene expression data from all participants in this study can be found publicly on the Gene Expression Omnibus (GEO) at www.ncbi.nlm.nih.gov/geo/ (accession number GSE162120).

## Supplementary Materials

The following are available online at https://www.mdpi.com/article/10.3390/biomedicines10051110/s1.
