# Supplementary material for: Inhaled Corticosteroids Selectively Alter the Microbiome and Host Transcriptome in the Small Airways of Patients with Chronic Obstructive Pulmonary Disease
Source: Biomedicines. 2022 May 11;10(5):1110. doi: 10.3390/biomedicines10051110 (PMC9138653; doi:10.3390/biomedicines10051110)
Supplement: Supplementary file 1 [file biomedicines-10-01110-s001.zip › biomedicines-1710674-supplementary.pdf]

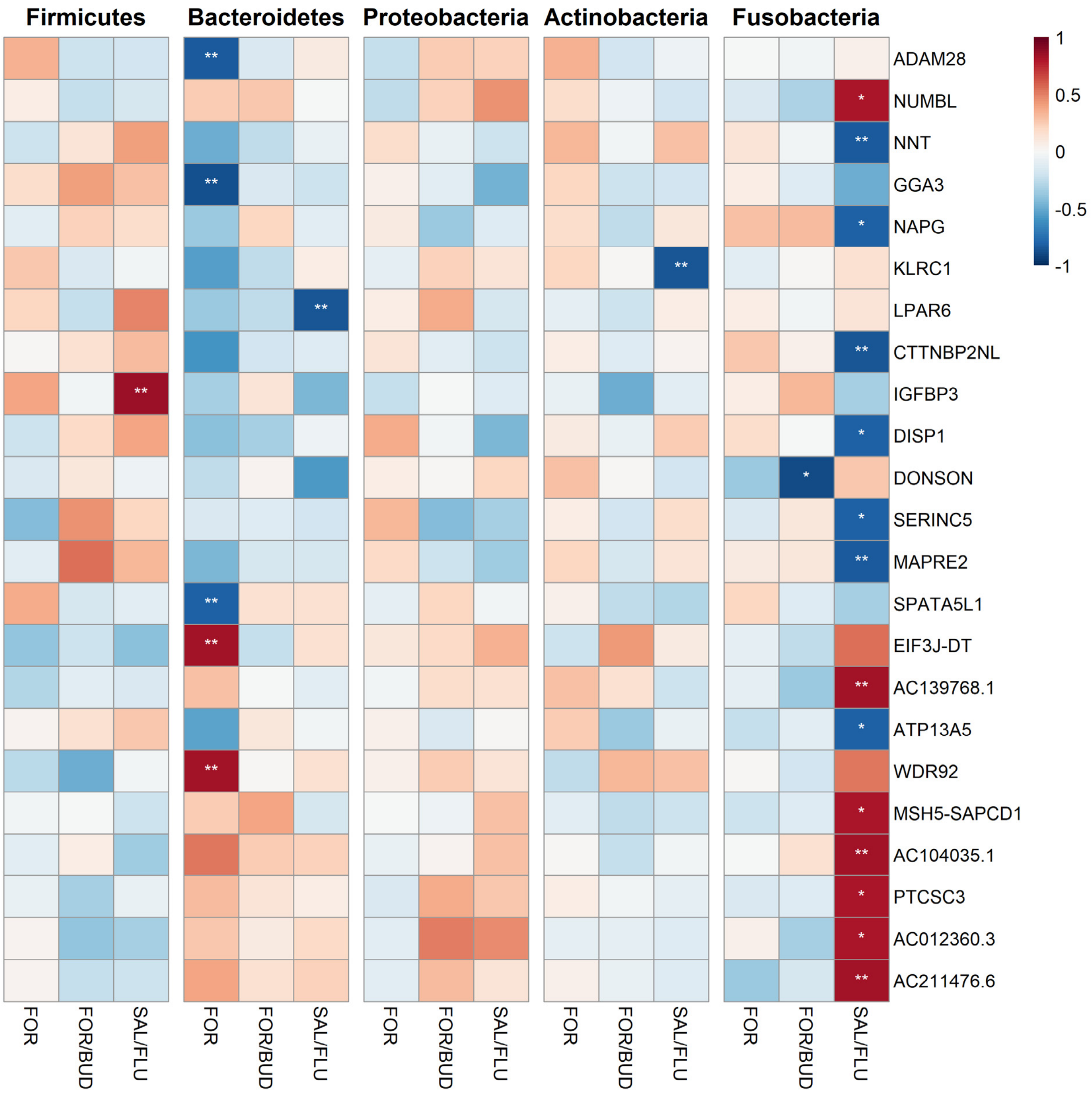

Figure S1. Heat map depicting significant correlations between changes (post- minus pre-treatment) in host cell gene expression (delta $\log_2$ counts per million [$\Delta\log_2$CPM]) and changes in the relative abundance of the top five most abundant phyla. Significantly correlated genes in at least one of the three treatment groups were reported. Colour represents the Spearman's correlation coefficient. Rows represent individual genes. ** represents a false discovery rate (FDR) < 0·05. * represents an FDR < 0·1. FOR = Formoterol-only; FOR/BUD = formoterol/budesonide; SAL/FLU = salmeterol/fluticasone.

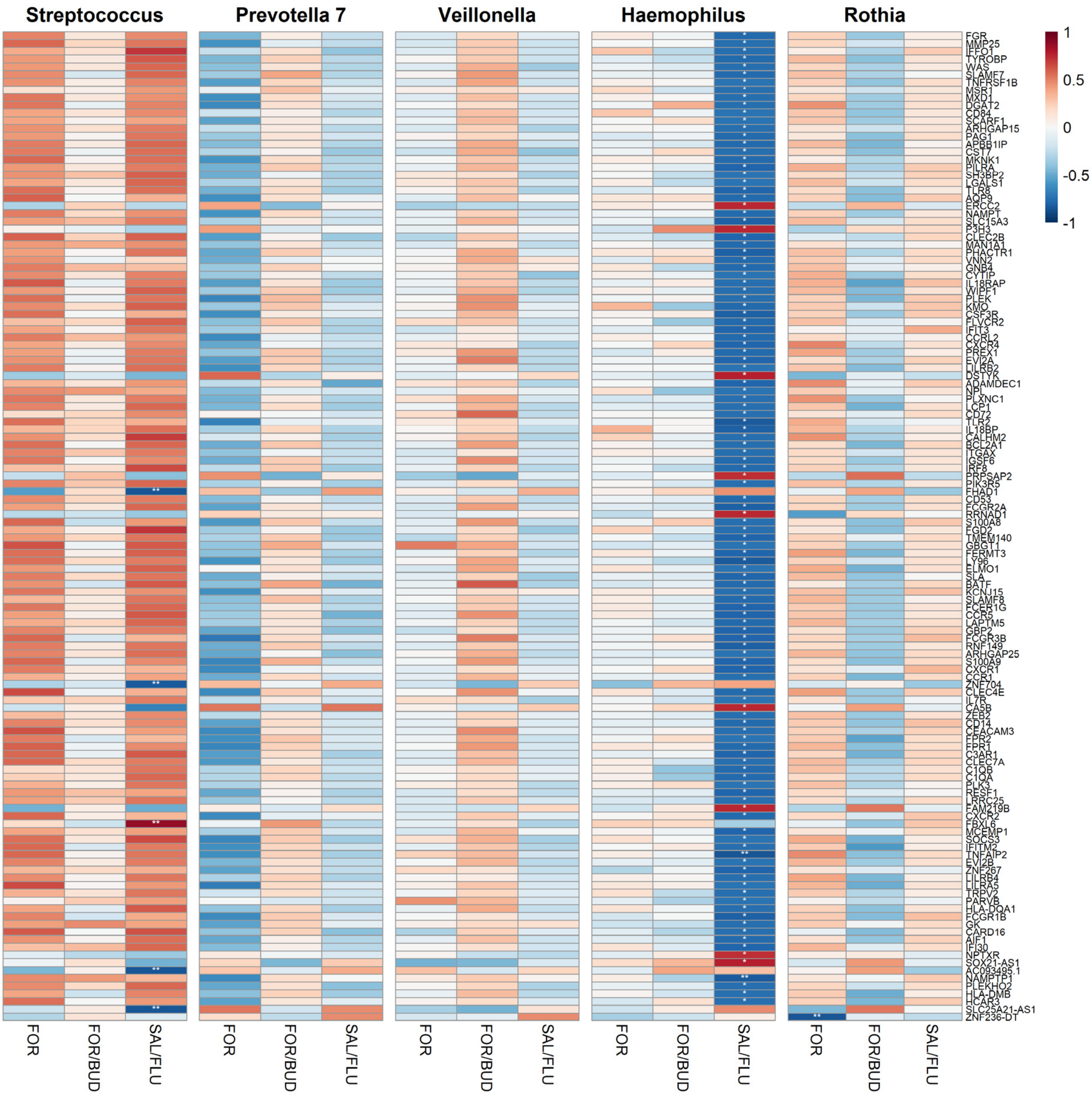

Figure S2. Heat map depicting significant correlations between changes (post- minus pre-treatment) in host cell gene expression (delta $\log_2$ counts per million [$\Delta\log_2$CPM]) and changes in the relative abundance of the top five most abundant genera. Significantly correlated genes in at least one of the three treatment groups were reported. Colour represents the Spearman's correlation coefficient. Rows represent individual genes. ** represents a false discovery rate (FDR) < 0·05. * represents an FDR < 0·1. FOR = Formoterol-only; FOR/BUD = formoterol/budesonide; SAL/FLU = salmeterol/fluticasone.

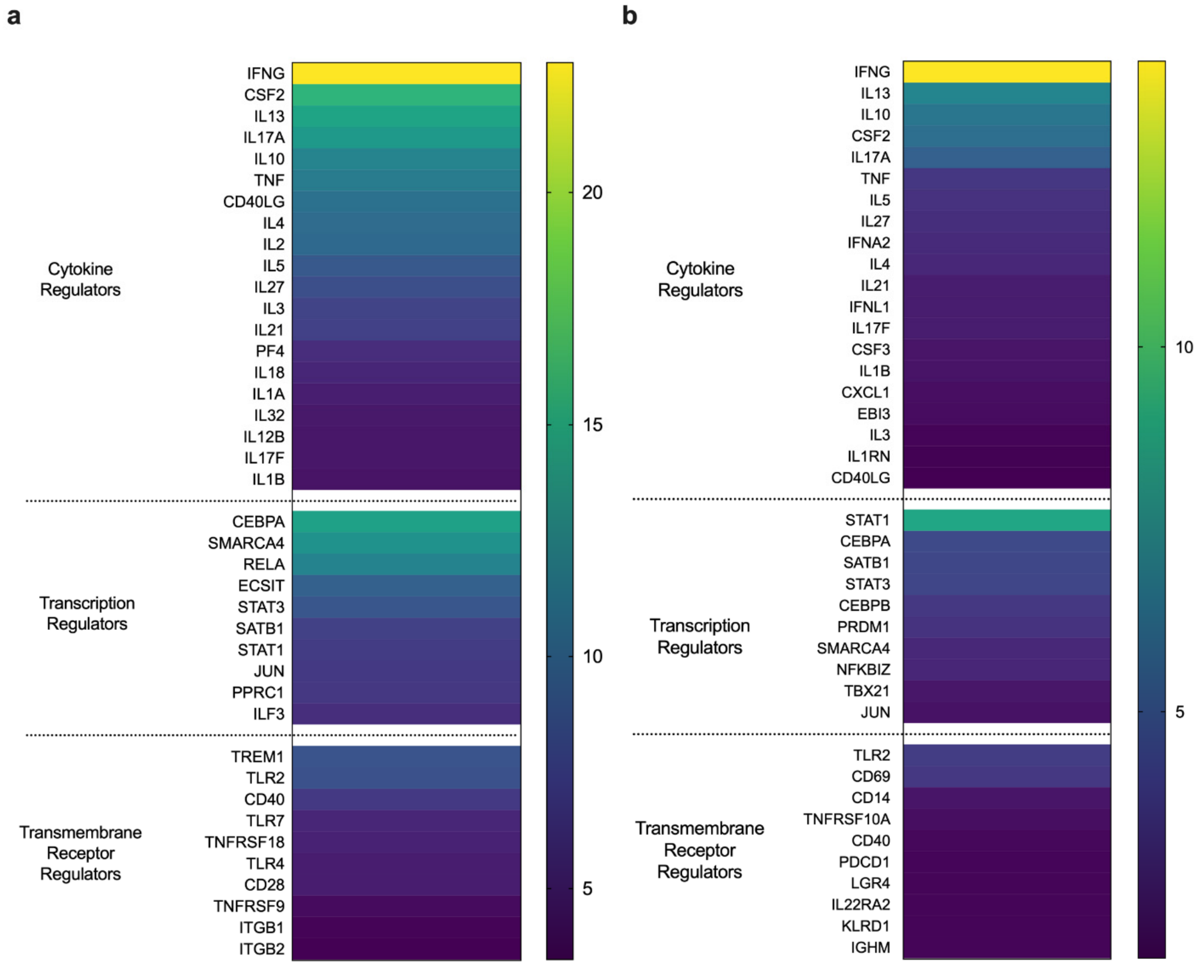

Figure S3. Ingenuity Pathway Analysis of predicted upstream transcription, cytokine and transmembrane receptor regulators. **A.** Heatmap of predicted upstream regulators of host cell genes significantly correlated to changes in the Shannon diversity index. **B.** Heatmap of predicted upstream regulators of host cell genes significantly correlated to changes in the relative abundance of Haemophilus. Rows represent individual predicted regulators. Colour represents the -log(p-value of overlap).

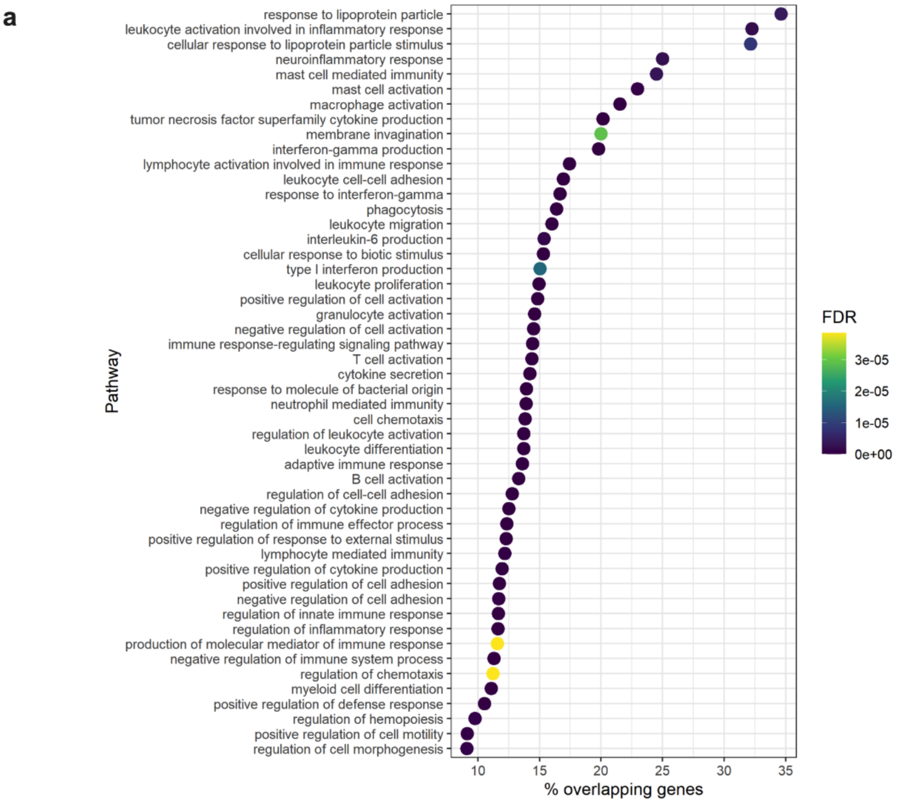

a
response to lipoprotein particle
leukocyte activation involved in inflammatory response
cellular response to lipoprotein particle stimulus
neuroinflammatory response
mast cell mediated immunity
mast cell activation
macrophage activation
tumor necrosis factor superfamily cytokine production
membrane invagination
interferon-gamma production
lymphocyte activation involved in immune response
leukocyte cell-cell adhesion
response to interferon-gamma
phagocytosis
leukocyte migration
interleukin-6 production
cellular response to biotic stimulus
type I interferon production
leukocyte proliferation
positive regulation of cell activation
granulocyte activation
negative regulation of cell activation
immune response-regulating signaling pathway
T cell activation
cytokine secretion
response to molecule of bacterial origin
neutrophil mediated immunity
cell chemotaxis
regulation of leukocyte activation
leukocyte differentiation
adaptive immune response
B cell activation
regulation of cell-cell adhesion
negative regulation of cytokine production
regulation of immune effector process
positive regulation of response to external stimulus
lymphocyte mediated immunity
positive regulation of cytokine production
positive regulation of cell adhesion
negative regulation of cell adhesion
regulation of innate immune response
regulation of inflammatory response
production of molecular mediator of immune response
negative regulation of immune system process
regulation of chemotaxis
myeloid cell differentiation
positive regulation of defense response
regulation of hemopoiesis
positive regulation of cell motility
regulation of cell morphogenesis
Pathway
10 15 20 25 30 35
% overlapping genes
FDR
3e-05
2e-05
1e-05
0e+00

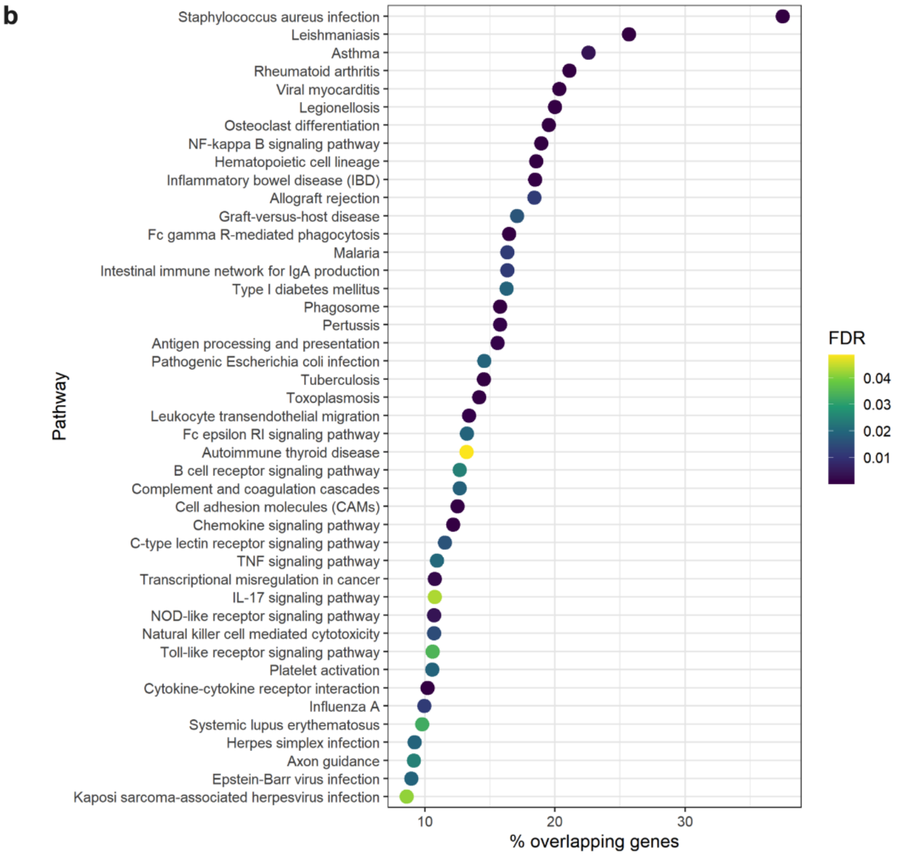

b
Staphylococcus aureus infection
Leishmaniasis
Asthma
Rheumatoid arthritis
Viral myocarditis
Legionellosis
Osteoclast differentiation
NF-kappa B signaling pathway
Hematopoietic cell lineage
Inflammatory bowel disease (IBD)
Allograft rejection
Graft-versus-host disease
Fc gamma R-mediated phagocytosis
Malaria
Intestinal immune network for IgA production
Type I diabetes mellitus
Phagosome
Pertussis
Antigen processing and presentation
Pathogenic Escherichia coli infection
Tuberculosis
Toxoplasmosis
Leukocyte transendothelial migration
Fc epsilon RI signaling pathway
Autoimmune thyroid disease
B cell receptor signaling pathway
Complement and coagulation cascades
Cell adhesion molecules (CAMs)
Chemokine signaling pathway
C-type lectin receptor signaling pathway
TNF signaling pathway
Transcriptional misregulation in cancer
IL-17 signaling pathway
NOD-like receptor signaling pathway
Natural killer cell mediated cytotoxicity
Toll-like receptor signaling pathway
Platelet activation
Cytokine-cytokine receptor interaction
Influenza A
Systemic lupus erythematosus
Herpes simplex infection
Axon guidance
Epstein-Barr virus infection
Kaposi sarcoma-associated herpesvirus infection
Pathway
10 20 30
% overlapping genes
FDR
0.04
0.03
0.02
0.01

Figure S4. Biological processes and pathways enriched from 676 host cell genes significantly correlated with changes in Shannon diversity index. **A.** Gene Ontology (GO) biological process enrichment. **B.** Kyoto Encyclopedia of Genes and Genomes (KEGG) pathway enrichment. Up to 50 of the top GO processes or KEGG pathways by FDR are shown, ordered by % overlapping genes (proportion of genes in the GO process or KEGG pathway that also appear in the list of 676 genes). Abbreviations: FDR, false discovery rate.

a

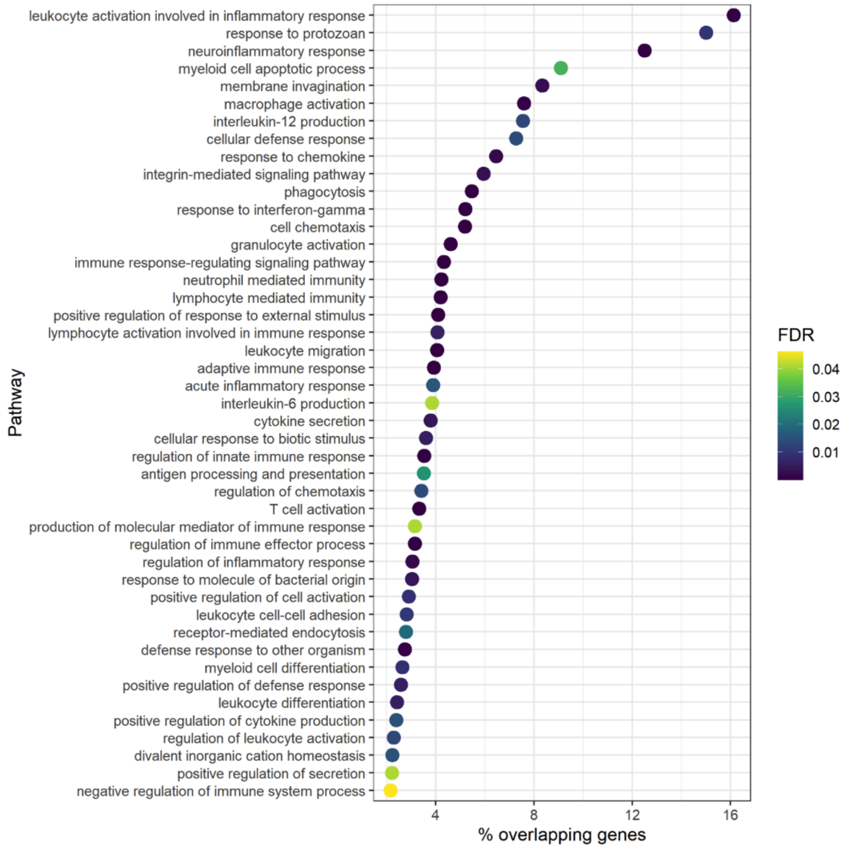

leukocyte activation involved in inflammatory response
response to protozoan
neuroinflammatory response
myeloid cell apoptotic process
membrane invagination
macrophage activation
interleukin-12 production
cellular defense response
response to chemokine
integrin-mediated signaling pathway
phagocytosis
response to interferon-gamma
cell chemotaxis
granulocyte activation
immune response-regulating signaling pathway
neutrophil mediated immunity
lymphocyte mediated immunity
positive regulation of response to external stimulus
lymphocyte activation involved in immune response
leukocyte migration
adaptive immune response
acute inflammatory response
interleukin-6 production
cytokine secretion
cellular response to biotic stimulus
regulation of innate immune response
antigen processing and presentation
regulation of chemotaxis
T cell activation
production of molecular mediator of immune response
regulation of immune effector process
regulation of inflammatory response
response to molecule of bacterial origin
positive regulation of cell activation
leukocyte cell-cell adhesion
receptor-mediated endocytosis
defense response to other organism
myeloid cell differentiation
positive regulation of defense response
leukocyte differentiation
positive regulation of cytokine production
regulation of leukocyte activation
divalent inorganic cation homeostasis
positive regulation of secretion
negative regulation of immune system process
Pathway
4
8
12
16
% overlapping genes
FDR
0.04
0.03
0.02
0.01

b

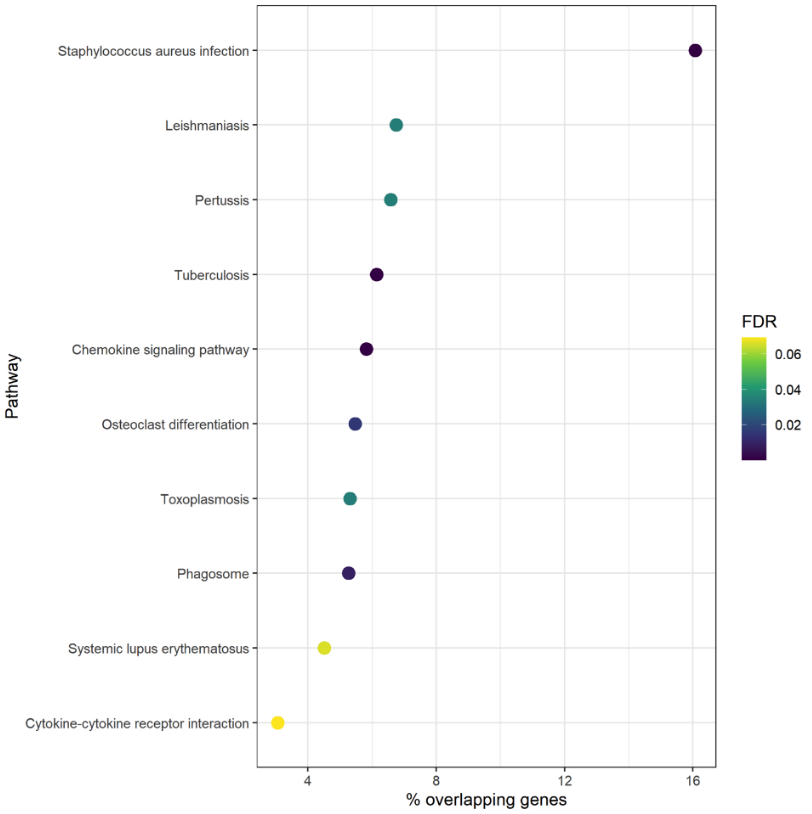

Staphylococcus aureus infection
Leishmaniasis
Pertussis
Tuberculosis
Chemokine signaling pathway
Osteoclast differentiation
Toxoplasmosis
Phagosome
Systemic lupus erythematosus
Cytokine-cytokine receptor interaction
Pathway
4
8
12
16
% overlapping genes
FDR
0.06
0.04
0.02

Figure S5. Biological processes and pathways enriched from 122 host cell genes significantly correlated with changes in the relative abundance of Haemophilus. **A.** Gene Ontology (GO) biological process enrichment. **B.** Kyoto Encyclopedia of Genes and Genomes (KEGG) pathway enrichment. Up to 50 of the top GO processes or KEGG pathways by FDR are shown, ordered by % overlapping genes (proportion of genes in the GO process or KEGG pathway that also appear in the list of 122 genes). Abbreviations: FDR, false discovery rate.
